# Transcriptional and Physiological Analysis Reveal New Insights into the Regulation of Fertilization (N, P, K) on the Growth and Synthesis of Medicinal Components of *Dendrobium denneanum*

**DOI:** 10.3390/ijms24021522

**Published:** 2023-01-12

**Authors:** Yijun Fan, Erya Xu, Gang Wang, Dingxin He, Jie Ma, Yuanyuan Liu, Xuebing Li, Aoxue Luo

**Affiliations:** 1 Department of Landscape Plants, Sichuan Agricultural University, Chengdu 611130, China; 2 College of Forest, Sichuan Agricultural University, Chengdu 611130, China

**Keywords:** *Dendrobium denneanum*, transcriptome, fertilization, orthogonal experimental design, medicinal compounds

## Abstract

*Dendrobium denneanum* is an important medicinal and ornamental plant. Its ornamental and medicinal values are affected by its vegetative growth conditions and chemical composition accumulation. This study adopted an orthogonal experimental design to treat *D. denneanum* with nine different levels of nitrogen (N), potassium (K), and phosphorus (P). The morphological indicators of the plant were positively correlated with the nitrogen concentration. The polysaccharide content was the highest at 1500 mg·L^−1^ nitrogen and 3000 mg·L^−1^ phosphorous and was 26.84% greater than the control. The flavonoid content increased by 36.2% at 500 mg·L^−1^ nitrogen, 2000 mg·L^−1^ phosphorous, and 300 mg·L^−1^ potassium. Principal component score analysis showed that nitrogen had the most significant impact on the various indicators of *D. denneanum*, followed by phosphorus and potassium. The comprehensive score showed that the T9 treatment (N: 1500 mg·L^−1^, P: 3000 mg·L^−1^, K: 500 mg·L^−1^) had the strongest effect on *D. denneanum*. Transcriptional analysis showed that compared with the control, the T9 treatment led to 2277 differentially expressed genes (1230 upregulated and 1047 downregulated). This includes fifteen genes enriched in the MAPK signaling pathway, five genes in phenylpropanoid biosynthesis, and two genes in flavonoid biosynthesis. These genes may be involved in regulating plant growth and the biosynthesis of polysaccharides and flavonoids. This study provides guidance for the optimal use of N, P, and K in the cultivation of *D. denneanum*.

## 1. Introduction

*Dendrobium* is a perennial herb of the family Orchidaceae. There are seventy-six species and two varieties of *Dendrobium* in China [1]; these include *D. denneanum, D. nobile, D. huoshan, D. candidum*, and *D. chrysotoxum*. As the second-largest genus of orchids, *Dendrobium* has high ornamental value and is one of the main four ornamental orchids. Moreover, *Dendrobium* is a valuable traditional Chinese medicine [2]. *D. denneanum* is the fresh or dry stem of *Dendrobium denneanum kerr*., a variety of *Dendrobium chryseum*, and mainly grows in mountains and trunks at an altitude of 600–2500 m. *D. denneanum* has a long history of application and rich resources in China, and has been included in the Pharmacopoeia of the People’s Republic of China. *D. denneanum* is rich in bioactive compounds, mainly including flavonoids [3], polysaccharides [4], phenanthrene glycosides [5], phenanthrenes [6], and Triterpene [7]. It has the functions of immunological enhancement [8], hypoglycemic [9], antioxidant [10,11], anti-tumor [12], anti-inflammatory [6], anti-osmotic [13], etc. The content of these chemicals determines the medicinal value of *Dendrobium* and can be used as an important index to evaluate its quality. The growth of *Dendrobium* and the synthesis and accumulation of medicinal compounds are affected by a variety of biotic and abiotic factors, such as genetics and environmental conditions [14,15].

During plant growth, in addition to the necessary light, water and oxygen, providing plants with appropriate nutritional fertilization promotes vegetative growth at all stages. Nitrogen, phosphorus, and potassium are the three most important elements for plant growth since plants have the largest demand for these elements, and they have the most apparent effect on plants [16]. These nutrients affect plant growth and the accumulation of chemical components. For example, Viola et al. [17] found that the N, P, and K treatment could increase the assimilation rate in *Centella asiatica*, improve leaf yield, and affect the accumulation of its main nutritional components. Sung et al. [18] found that the lack of N, P, or K decreased energy production and the metabolism of organic acids, soluble sugars, and amino acids in *Solanum lycopersicum L*. Deng et al. [19] treated *Ophiopogon japonicus* with different concentrations of fertilizer and found that the levels of N, P, and K mainly affected the contents of saponin D’, methyl flavanone A, and methyl flavanone B. Tang et al. [20] found that the nutrients required by each *Dendrobium* plant during its vegetative growth period were 1.67 mg nitrogen, 0.47 mg phosphorus, and 2.10 mg potassium. Therefore, during plant growth, the rational use of nitrogen, phosphorus, and potassium could promote its growth and improve its ornamental value and quality. Improper fertilization might damage the plant and affect its normal growth. Therefore, studying fertilization with different ratios of nitrogen, phosphorus, and potassium is important. *D. denneanum* is valued for its ornamental and medicinal qualities. However, no studies have investigated whether the concentrations of nitrogen, phosphorus, and potassium affect its growth, chemical components, and quality.

In this study, an orthogonal design was used to conduct a combination test of the nitrogen, phosphorus, and potassium fertilizer formulation. By analyzing morphological indicators and the contents of the main chemical components, the optimal ratio of nitrogen, phosphorus, and potassium for the growth of *D. denneanum* and for the accumulation of its main medicinally active components were elucidated. Additionally, the analysis of transcriptome revealed key-genes in the plant’s response to nitrogen, phosphorus, and potassium. The findings provide a rationale for the optimal fertilization regime and could be used to support plant management measures and the formulation of quality standards for the medicinal materials produced in cultivated *D. denneanum*.

## 2. Results

### 2.1. Effects of N, P, and K on the Morphological Characteristics

**Figure 1:**
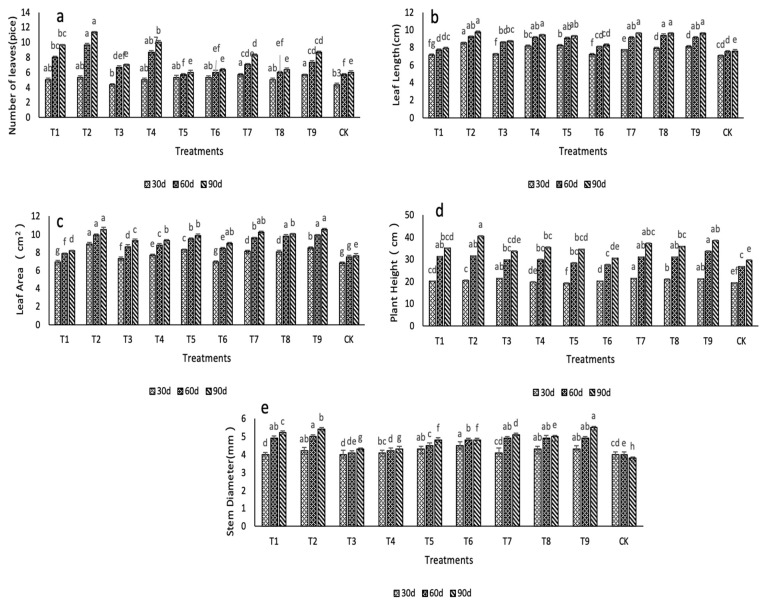
Effects of nitrogen, phosphorus, and potassium on the morphological characteristics of *D. denneanum*. (**a**) Number of leaves. (**b**) Leaf length. (**c**) Leaf area. (**d**) Plant height. (**e**) Stem diameter. Data are means ± SE (*n* = 5) in the figure. Different letters indicate significant differences among the treatments (*p* < 0.05).

At 30 d, the number of leaves in all treatment groups were increased compared with the control, except for the T3 treatment group. The number of leaves in the T9 treatment group increased by 30.95% compared with the ck (*p* < 0.05). At 60 d, the number of leaves in the treatment groups increased except for the T5 treatment group. Compared with the ck, the number of leaves in the T2, T4, T1, T9, and T7 treatment groups was greater by 70.55%, 52.91%, 41.09%, 29.28%, and 23.46%, respectively (*p* < 0.05). At 90 d, except for the T5 treatment group, the number of leaves in all other treatment groups significantly increased compared with the control (*p* < 0.05). The number of leaves in the T2 treatment group increased the most, with an increase of 88.33% (*p* < 0.05) (Figure 1a).

Potassium had a significant effect on the number of leaves as evidenced by the changes in the number of leaves in the treatment groups from 30 to 90 d. The largest difference in the number of leaves between the treatment and control groups occurred at the K2 concentration (*p* < 0.05), indicating that 500 mg·L^−1^ potassium effectively increased the number of leaves. The number of leaves in the changing nitrogen concentration rates was is the order of N1 > N3 > N2. No clear differences in leaf number were found upon changing the phosphorus application rate (*p* > 0.05).

Figure 1b shows that at 30 d, the leaf lengths in T2, T5, T4, and T9 were greater than the control, increasing by 21.19%, 17.07%, 15.65%, and 14.79%, respectively. At 60 d, the T8 treatment increased leaf length by 24.60% compared with the control, and the T2, T9, T7, T4, and T5 treatments increased it by 22.34%, 21.41%, 21.01%, 21.01%, and 20.35%, respectively. At 90 d, the T2, T7, T9, T8, and T4 treatments increased the leaf length by 28.53%, 27.08%, 26.82%, 26.55%, and 24.44%, respectively.

With the increase in nitrogen application concentration, the leaf length increased in the order of N3 > N2 > N1. The leaf length was significantly increased in the N3 treatment group, indicating that a nitrogen concentration of 1500 mg·L^−1^ promoted an increase in leaf length. In addition, under this nitrogen concentration, the leaf length was significantly increased when the potassium concentration was 500 mg·L^−1^. The T2 treatment (500 mg·L^−1^ nitrogen, 2000 mg·L^−1^ phosphorus, and 500 mg·L^−1^ potassium) resulted in the largest leaf length and the highest growth rate.

Figure 1c shows that with increasing nitrogen application concentration, the maximum leaf area increased in the order of N3 > N2 > N1. The maximum leaf area was significantly larger than the control in the T7, T8, T9, and T2 treatment groups. At 90 d, the difference in the maximum leaf area between the treatment groups and the control group was the largest. The T9 treatment increased the leaf area by 38.58%, the T2 treatment increased it by 38.45%, the T7 treatment increased it by 33.86%, and the T8 treatment increased it by 32.55%. Therefore, 1500 mg·L^−1^ nitrogen, 2000 mg·L^−1^ phosphorus, and 500 mg·L^−1^ potassium had a positive effect on the maximum leaf area.

Figure 1d shows that with increasing nitrogen concentration, the plant height increased in the order of N3 > N1 > N2. At 30 d, the plant heights of the T7 and T3 treatment groups were 10.59% higher than the ck (*p* < 0.05). At 60 d, the plant height of T9 treatment was 26.10% higher than the ck. At 90 d, the plant heights of the T2, T1, T7, and T8 treatment groups were 18.24%, 17.34%, 16.10%, and 16.10% higher (*p* < 0.05) than the ck. Among them, T2 treatment increased plant height the most, resulting in plants that were 36.10% higher than the control. The increase in plant height in each treatment group was greatest between 30 and 60 days, while the increase in plant height lessened between 60 and 90 days, which was consistent with the trends observed for the number of leaves and maximum leaf area. The treatment that promoted plant height the most was 500 mg·L^−1^ nitrogen, 2000 mg·L^−1^ phosphorus, and 500 mg·L^−1^ potassium.

Figure 1e shows that at 30 d, the T6 group had the largest increase in stem diameter and was 12.50% larger than the control. At 60 d, the stem diameter increased the most in the T2 treatment and was 25.00% larger than the control (*p* < 0.05), followed by the T1 treatment, which was 22.50% (*p* < 0.05) larger than the ck. At 90 d, the largest increase in stem diameter was observed in the T9 treatment, which was 44.74% larger than the control (*p* < 0.05).

### 2.2. Effects of N, P, and K on the Physiological Characteristics of D. denneanum

Table 1 shows that at 30 d, all treatment groups (except for T1 and T3) were significantly different compared with the ck (*p* < 0.05). T2 had the highest chlorophyll A content in the N1 treatment, which was 13.33% (*p* < 0.05) higher than the ck. T5 had the highest chlorophyll A content in the N2 group, which was 15.56% (*p* < 0.05) higher than the ck. Among them, the T7, T8, and T9 treatments resulted in chlorophyll A contents that were 26.67%, 22.22%, and 24.44% higher than the ck (*p* < 0.05), respectively. At 60 d, there were significant differences between each treatment and the control (*p* < 0.05). In the N1 treatment, T2 had the highest chlorophyll A content, which was 15.28% higher than the control, while in T5, the N2 treatment had a chlorophyll A content that was 12.50% higher than the control (*p* < 0.05). In the N3 treatment, chlorophyll A was 19.44%, 18.06%, and 20.83% higher than the control (*p* < 0.05), respectively. At 90 d, the chlorophyll A content in each treatment was significantly higher than the ck. These results indicate that nitrogen was the main factor affecting chlorophyll A content.

The changing trend of the chlorophyll B content was similar to chlorophyll A. At 30 d, T2 had the highest chlorophyll B content in the N1 treatment, which was 13.04% higher than the ck. T5 had the highest chlorophyll B content in the N2 treatment, which was 30.43% higher than the control (*p* < 0.05). T7, T8, and T9 in the N3 treatment group had chlorophyll B contents that were 26.09%, 26.09%, and 30.43% higher than the ck (*p* < 0.05), respectively. At 60 d, all treatments except for T1, T3, and T6 had significantly increased chlorophyll B contents compared with the control (*p* < 0.05), among which T2 had the largest increase, at 15.38% higher than the ck. At 90 d, the content of chlorophyll B in T8 treatment was the highest and was 17.50% higher than the control. The T2, T9, T7, and T4 treatments were 15.00%, 15.00%, 10.00%, and 10.00% higher than the control, respectively (*p* < 0.05). During treatment, the chlorophyll A and chlorophyll B contents gradually increased. However, at 90 d, these contents in the control group were increased by less than 3% compared with the 60 d values, while the other treatment groups showed significant increases, indicating that spraying nitrogen, phosphorus, and potassium foliar fertilizer was conducive to increasing the chlorophyll content in the leaves. Furthermore, with the increase in nitrogen concentration, the chlorophyll content of plant leaves increased.

**Figure 2:**
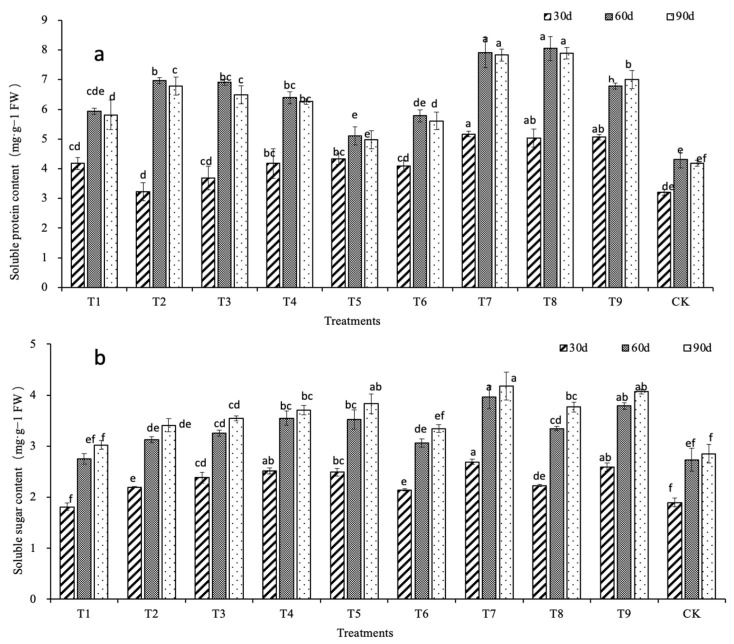
Contents of soluble protein and soluble sugar. (**a**) Soluble protein content. (**b**) Soluble sugar content. Lowercase letters indicate inter group significance (*p* < 0.05).

The result shows that the content of soluble protein initially increased and then decreased (Figure 2a). At 30 d, with the increase in nitrogen application concentration, the soluble protein content in the N3 treatment increased the most compared with the control, and in the T7 treatment it was 87.54% higher than the control (*p* < 0.05). The soluble protein content of the N2 treatment was slightly higher than the N1 treatment (*p* > 0.05). At 60 d, except for T5 and T6 treatments, there were significant differences between all other treatments and the control (*p* < 0.05). Among them, the T7, T8, and T9 treatments were 64.45%, 69.44%, and 43.66% higher than the control, respectively. At 90 d, the content of soluble protein in N3 treatment decreased the least compared with the other treatments, and the T8 treatment increased the most significantly compared with the control (*p* < 0.05). Among them, the T7, T8, and T9 treatments were 76.31%, 82.69%, and 57.18% higher than the control, respectively (*p* < 0.05). Except for the T5 treatment, the soluble protein content of plant seedlings in the other treatments increased initially and then decreased with increasing phosphorus concentration under the same nitrogen concentration, indicating that phosphorus can affect the soluble protein content. At 90 d, the soluble protein content was in the order of N3 > N1 > N2, and the P2 concentration gave the largest soluble protein content. The soluble protein content in the N3 treatment group was the maximum in each month, indicating that a nitrogen concentration of 1500 mg·L^−1^ was the most conducive to promoting the synthesis of soluble protein in the *D. denneanum* leaves.

Figure 2b shows that the soluble sugar content gradually increased with the growth of *D. denneanum* stem. At 30 d, the soluble sugar contents of T7 and T9 in the N3 treatment were higher by 41.58% and 36.32%, respectively, than the control (*p* < 0.05). In the N1 treatment, the soluble sugar contents of the T2 and T3 treatments were significantly increased compared with the control (*p* < 0.05) and were higher by 15.79% and 25.26%, respectively. At 60 d, the soluble protein content of each treatment was significantly increased compared with the content at 30 d. In N3 treatment, the T7, T8, and T9 treatments were increased by 47.21%, 50.22%, and 46.33%, respectively, compared with the soluble protein content at 30 d. The overall growth rate of the soluble sugar content in the stem of *D. denneanum* increased with increasing nitrogen application concentration. Under the same nitrogen concentration, there was a positive correlation between the soluble sugar content and potassium concentration. At 90 d, except for the T1 and T6 treatments, the soluble sugar contents of all other treatments were significantly different from the control (*p* < 0.05), and the change trend was consistent with the content at 60 d. The results showed that a suitable concentration of nitrogen, phosphorus, and potassium could significantly increase the soluble sugar content in the plants. At the same nitrogen application rate, a higher potassium concentration could increase the soluble sugar content in the *D. denneanum* stems.

**Figure 3:**
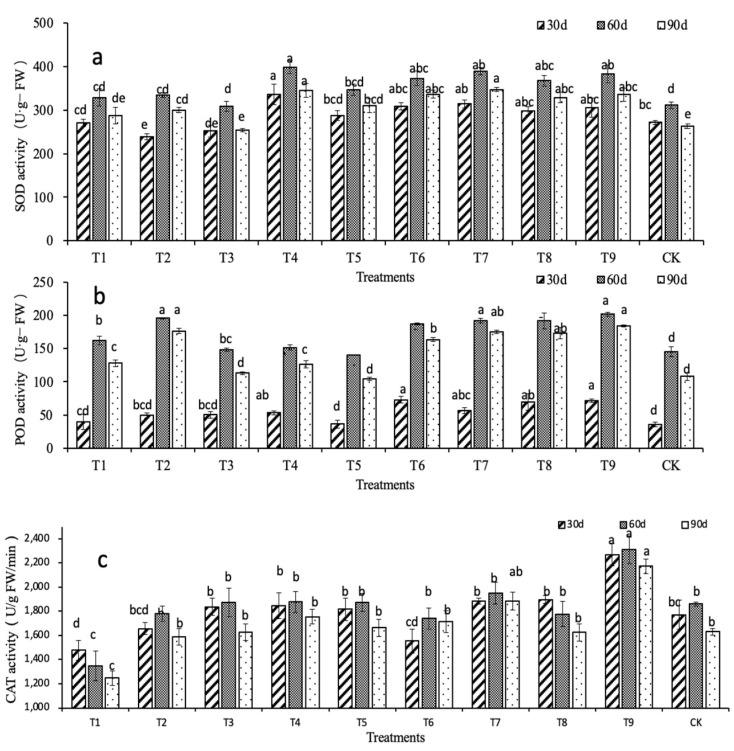
Effects of nitrogen, phosphorus, and potassium on the antioxidant system activity of *D. denneanum*. (**a**) SOD activity. (**b**) POD activity. (**c**) CAT activity. Lowercase letters indicate inter group significance (*p* < 0.05).

Figure 3a shows that the superoxide dismutase (SOD) activity increased initially and then decreased. At 30 d, except for the T1, T2, and T3 treatments at the N1 concentration, the SOD activity of the other treatments was significantly higher than the control (*p* < 0.05). The most significant difference was in the T4 treatment, which was 23.55% higher than the ck. The second-largest difference was seen in the T7 treatment, which was 15.51% higher than the ck. At 60 d, the SOD activity was increased compared with the activity at 30 d, but the increases were different. Among them, the largest increase was in the T2 treatment, with an increase of 40.13%. At 60 d, the SOD activity peaked in T4 at 28.00% higher than the ck. The increases in the T7 and T9 treatments were also significant (*p* < 0.05), at 25.02% and 22.89% higher than the ck, respectively. At 90 d, the SOD activity in each treatment group was decreased to varying degrees. The results show that a high nitrogen concentration more effectively promotes SOD activity than a low nitrogen concentration.

Figure 3b shows that the peroxidase (POD) activity in *D. denneanum* initially increased and then decreased. POD activities in the T6, T7, T8, and T9 treatments were significantly higher than the control (*p* < 0.05) and were higher by 102.77%, 58.33%, 93.53%, and 99.08%, respectively at 30 d. At 60 d, the POD activity in each treatment group was sharply increased and was highest in the T9 treatment, which was 38.44% higher than the ck. POD activity had decreased to varying degrees at 90 d.

Figure 3c shows that there were large differences in catalase (CAT) activity among the treatment groups. The CAT activity in T1 and T8 decreased, but the CAT activity in the other treatments and control increased initially and then decreased. At 30 d, the lowest CAT activity was in the T1 treatment, which was 16.57% lower than the control (*p* < 0.05). The highest CAT activity was in the T9 treatment, which was 28.25% higher than the control (*p* < 0.05). CAT activities of the T1 and T8 treatments were 27.42% and 4.52% lower than the control at 60 d, respectively. The CAT activity of the T9 treatment was the highest and was 24.19% higher than the control. CAT activity of each treatment and control had decreased to varying degrees at 90 d. The CAT activity of the T1 treatment was the lowest. Compared with the other treatments, the T9 treatment maintained the highest level, which was 33.33% higher than the control. Therefore, the T9 treatment (N3 = 1500 mg·L^−1^, P3 = 3000 mg·L^−1^, K2 = 500 mg·L^−1^) had the most apparent effect on improving the CAT activity in *D. denneanum*.

### 2.3. Effects of N, P, and K on the Main Medicinal Compounds

**Figure 4:**
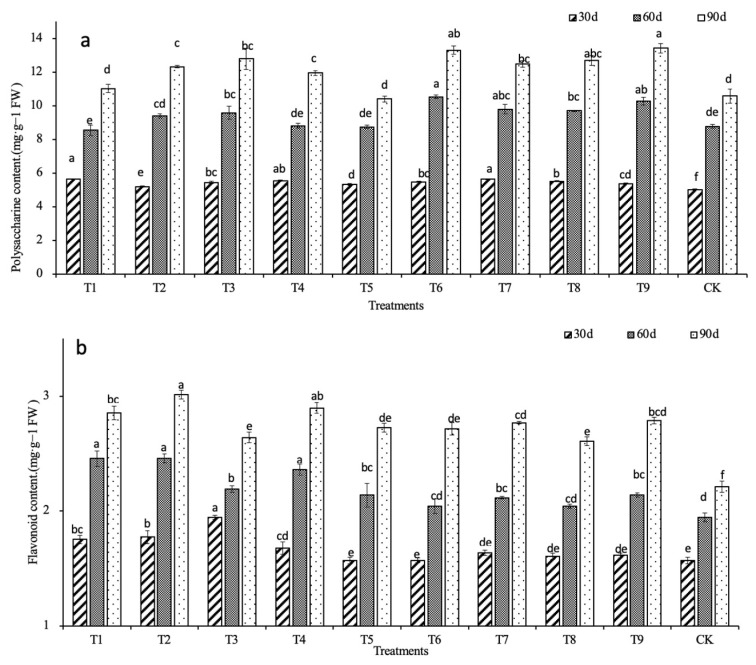
Effects on the content of main medicinal compounds. (**a**) Polysaccharide content. (**b**) Flavonoids content. Lowercase letters indicate inter group significance (*p* < 0.05).

The accumulation of polysaccharides in the *D. denneanum* seedlings was slow at 30 d. Only the T1 and T7 treatments showed a significant increase compared with the control (*p* < 0.05). At 60 d, except for the T1, T2, T4, and T5 treatments, the polysaccharide contents of the other treatments were significantly increased compared with the control (*p* < 0.05), among which the T6 and T9 treatments increased the most. At 90 d, except for the T1 and T5 treatments, the polysaccharide contents of the other treatments were significantly higher than the control (*p* < 0.05). The polysaccharide contents of the T6 and T9 treatments were 25.71% and 26.84% higher than the control, respectively. The polysaccharide content of the T9 treatment peaked at 13.42 mg·g^−1^. The T6 and T9 treatments were most conducive to polysaccharide accumulation in *D. denneanum* (T6 = 1000 mg·L^−1^ nitrogen, 3000 mg·L^−1^ phosphorus, 300 mg·L^−1^ potassium; T9 = 1500 mg·L^−1^ nitrogen, 3000 mg·L^−1^ phosphorus, 500 mg·L^−1^ potassium) (Figure 4a).

During the growth of *D. denneanum*, the content of flavonoids showed a continuous upward trend. At 30 d, there was no significant difference in flavonoid content between any treatment group and the control. However, over time, the flavonoid content in each treatment group became significantly higher than the control. At 30 d, the flavonoid content in the T3 treatment group was the highest and was 24.20% higher than the control. The flavonoid content in each treatment group had increased to varying degrees at 60 d. Compared with the control, the largest increase was in the T1, T2, and T4 treatments, which increased by 26.15%, 26.15%, and 21.03%, respectively. At 90 d, the flavonoid contents were increased compared with those at 60 d and were significantly higher than the control (*p* < 0.05). Compared with the control, T1, T2, and T4 treatments remained at a high level, increasing by 28.96%, 36.20%, and 31.22%, respectively. At 60 d, the T2 treatment had the highest flavonoid content (3.01 mg·g^−1^) (Figure 4b).

The changes in flavonoid contents in the different treatment groups showed no clear differences between treatments 30 days after fertilizing. However, the flavonoid contents gradually increased in the later stage as growth proceeded and was finally significantly different from the control. These results indicate that the nitrogen, phosphorus, and potassium fertilizer increased the flavonoid content of *D. denneanum*.

### 2.4. Correlation Analysis

Table 2 presents the correlations between the morphological characteristics, physiological characteristics, and main medicinal compounds in the nitrogen, phosphorus, and potassium treatment groups at 90 d. There was a positive correlation between the number of leaves and the flavonoid content and plant height. The maximum leaf area showed a strong positive correlation with the plant height, chlorophyll A content, chlorophyll B content, and soluble sugar content (*p* < 0.01), along with a strong positive correlation with the stem diameter, soluble protein content, and POD activity (*p* < 0.05). Plant height showed a strong positive correlation with stem diameter, chlorophyll A content, and chlorophyll B content (*p* < 0.01), along with a strong positive correlation with soluble protein content and flavonoid content (*p* < 0.05). There was a strong positive correlation between the stem diameter and chlorophyll A content (*p* < 0.01) and between POD activity and flavonoid content (*p* < 0.05). There was a strong positive correlation between the chlorophyll A content and chlorophyll B content (*p* < 0.01) and a strong positive correlation between the soluble protein content, soluble sugar content, POD activity, and SOD activity (*p* < 0.01). The chlorophyll B content showed a strong positive correlation with the soluble protein content, soluble sugar content, and POD activity (*p* < 0.05). The soluble protein content showed a strong positive correlation with the soluble sugar content, POD activity, and polysaccharide content (*p* < 0.05). There was a strong positive correlation between the soluble sugar content and the SOD and CAT activities (*p* < 0.05). There was a strong positive correlation between the POD activity and the polysaccharide content (*p* < 0.01). No correlations were observed for the other indicators.

### 2.5. Principal Component Analysis

In this analysis, the original test data were standardized by the Z score method using SPSS 27 software, and the applicability of the experimental data was tested by the KMO method and Bartlett’s sphericity test method. The KMO value was 0.662, indicating that the indices of this test were related to each other. In addition, the significance value was 0.000, indicating that the indicators of the matrix were related. These test results showed that the test data could be used in the principal component analysis.

Table S1 presents the score coefficients of physiological, antioxidant system enzyme activity, and secondary metabolite content indices in *D. denneanum* under the different nitrogen, phosphorus, and potassium treatments. In the table, the scores of each index for two different parameters represent the load coefficient. In the first component, the score coefficient of chlorophyll B was the highest (0.161), and the score coefficient of flavonoids was the lowest (0.102). In the second component, the score coefficient of CAT was the highest (0.605), and the score coefficient of flavonoids was the lowest, as in the first component. As shown in Table 3, after standardization using the Z score method, each index had a load coefficient for both the first component and the second component (Figure S1). Therefore, the comprehensive score of each treatment could be analyzed through these two components.

Table 3 shows the principal component score and the comprehensive score of each treatment and control. The higher the score, the stronger the effect of the nitrogen, phosphorus, and potassium levels of the treatment on the various indices of *D. denneanum*. In the first component, the highest score was for the T9 treatment, followed by the T7 treatment, and the lowest score was for the CK treatment. In the second component, the highest score was also for the T9 treatment, followed by the CK treatment, and the lowest was for the T1 treatment. Regarding the score distribution and comprehensive score of these two components, with the increase in nitrogen application, the score of each treatment gradually increased as a whole, indicating that the nitrogen concentration had an apparent effect on the various indices of *D. denneanum*, and the effect was stronger with increasing nitrogen concentration. In addition, the first component and the comprehensive score showed that at the same nitrogen level, the K2 level also had a strong effect on each index. The K2 concentration was the most suitable potassium concentration in this experiment, and higher and lower potassium concentrations could not achieve the optimal effect. The effect of phosphorus and potassium on the various indices was less apparent than nitrogen, which may be related to the different varieties, growth periods, substrates, or environments of Dendrobium. The comprehensive score showed that the T9 treatment (1500 mg·L^−1^ nitrogen, 3000 mg·L^−1^ phosphorous, and 500 mg·L^−1^ potassium) had the strongest effect on *D. denneanum* in this experiment.

### 2.6. Transcriptome Sequencing Results and Data Assembly

A total of 405,988,182 raw reads and 61,304,215,482 raw bases (Table S2) were produced by transcriptome sequencing of *D. denneanum*. After removing the sequencing joints and low-quality raw reads and raw bases, a total of 390,094,696 high-quality clean reads were obtained, and 56,958,027,778 clean bases were generated. The percentage of Q20 bases was 97.65% or more, the percentage of Q30 bases was 93.35% or more, the average error rate was 0.02538%, and the percent GC content was between 44.75% and 45.76%. These results indicate that the transcriptome sequencing results had high base quality, a low error rate, and good sequencing quality; therefore, they could be used for subsequent analysis.

### 2.7. Differential Gene Analysis Expression of D. denneanum

**Figure 5:**
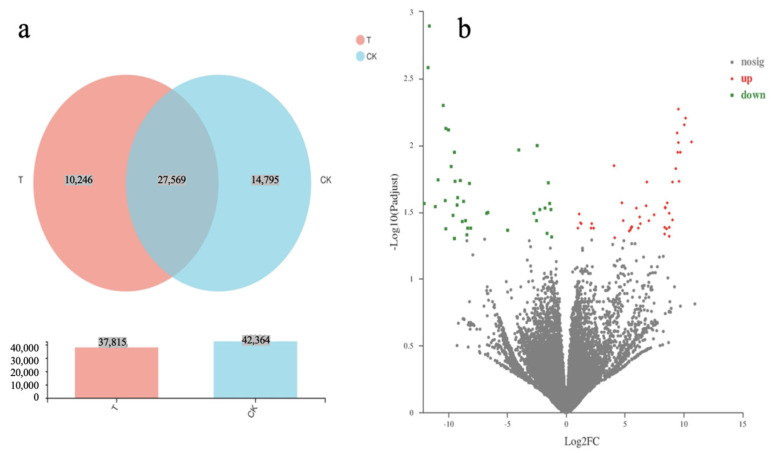
The differential gene analysis expression of T9 on *D. denneanum.* (**a**) Venn diagram. (**b**) Volcano plot. The *q*-value < 0.05 and |log_2_(fold change)| > 1 was used as the screening condition for differential expression.

The differentially expressed genes in the CK and T9 groups are shown in Figure 5. A total of 2277 differentially expressed genes were identified by comparing the CK and T9 treatments using a volcano map (Figure 5b), including 1230 upregulated and 1047 downregulated genes. A Wayne diagram (Figure 5a) was used to show the differentially expressed genes of the two combinations, of which 27,569 genes were expressed in both combinations, 10,246 genes were expressed in the T treatment, and 14,795 genes were expressed in the CK comparison.

### 2.8. GO Functional Enrichment Analysis

**Figure 6:**
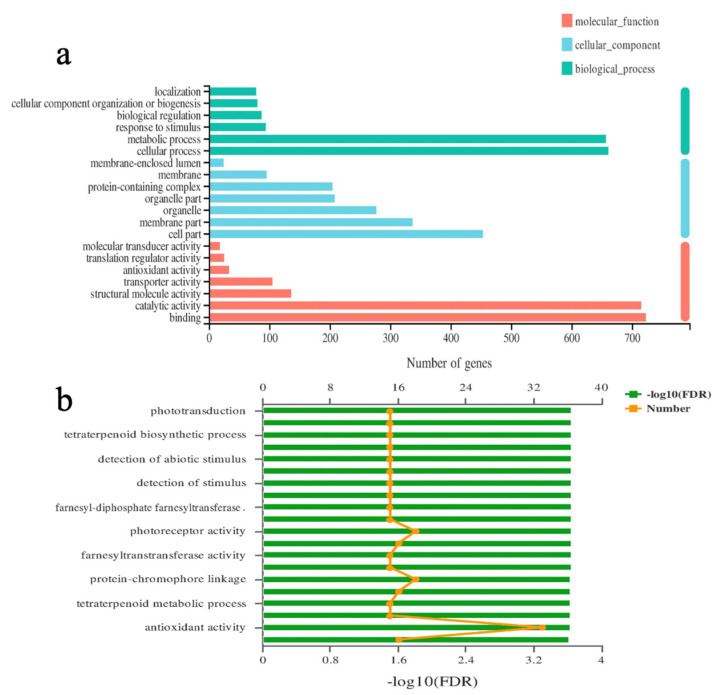
Bubble diagram of GO functional enrichment results of differentially expressed genes in the T9 treatment. (**a**) Significantly enriched the Top 10 GO terms. (**b**) Difference genes with the most significant enrichment degree.

Figure 6a shows the top 10 most significantly enriched GO terms. In the molecular function category, the terms were mainly enriched in the following seven functions: Molecular translator activity, translation regulator activity, antioxidant activity, transporter activity, structural molecular activity, catalytic activity, and binding. The cell component category was mainly enriched in the following seven functions: Cell part, membrane part, organelle, organelle part, protein containing complex, membrane, and membrane enclosed lumen. Biological process was mainly enriched in the following six functions: Localization, cellular component organization or biogenesis, biological regulation, response to stimulus, metabolic process, and cellular process.

GO enrichment analysis was further carried out on the differentially expressed genes, and the results showed a distribution of biological processes, cell components, and molecular functions. Ten GO entries with the most significant enrichment were selected for display (Figure 6b). The results showed that treatment Group (T9) was significantly enriched in phototransduction, tetraterpenoid biosynthetic process, detection of abiotic stimulus, detection of stimulus, farnesyl-diphosphate farnesyltransferase activity, photoreceptor activity, farnesyltransferase activity, protein-chromophore linkage, tetraterpenoid metabolic process, and antioxidant activity. Notably, treatment Group (T9) was more significantly enriched in four GO terms: Photoreceptor activity, protein chromophore linkage, and antioxidant activity.

### 2.9. KEGG Functional Enrichment Analysis

**Figure 7:**
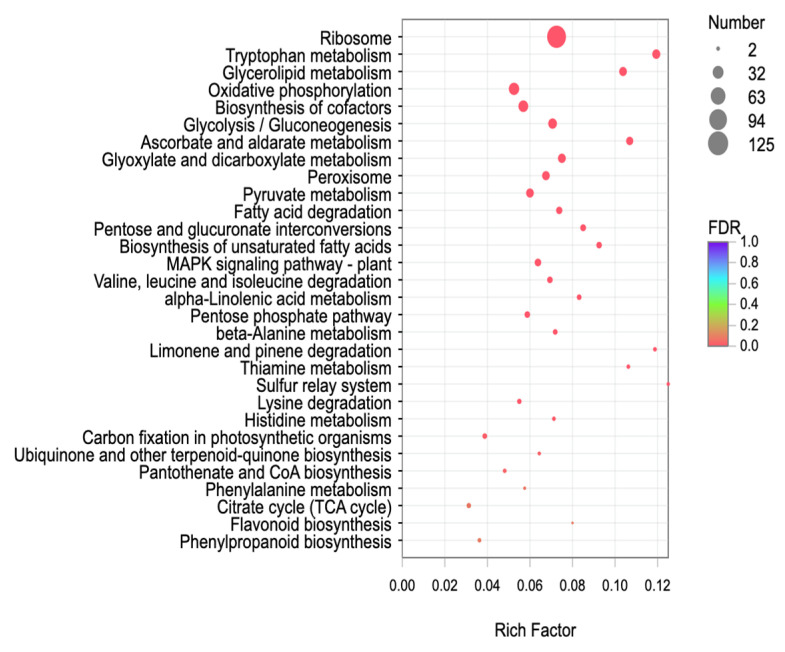
Classification of the top 30 enriched KEGG pathways of the differentially expressed genes between T9 and CK.

KEGG enrichment analysis was also carried out for the differentially expressed genes. The results are shown in Figure 7. Treatment Group (T9) was significantly enriched in the following 30 metabolic pathways: Ribosome, tryptophan metabolism, glycerolipid metabolism, oxidative phosphorylation, biosynthesis of cofactors, glycolysis/gluconeogenesis, ascorbate and aldarate metabolism, glyoxylate and dicarboxylate metabolism, peroxisome, pyruvate metabolism, fatty acid degradation, pentose and glucuronate interconversions, biosynthesis of unsaturated fatty acids, MAPK signaling pathway-plant, valine, leucine and isoleucine degradation, alpha-linolenic acid metabolism, pentose phosphate pathway, beta-alanine metabolism, limonene and pinene degradation, thiamine metabolism, sulfur relay system, lysine degradation, histidine metabolism, carbon fixation in photosynthetic organisms, ubiquinone and other terpenoid quinone biosynthesis, pantothenate and CoA biosynthesis, phenylalanine metabolism, citrate cycle (TCA cycle), flavonoid biosynthesis, and phenylpropanoid biosynthesis. The number of genes in the ribosome pathway was the largest, and the numbers of genes in the phenylpropanoid biosynthesis, phenylalanine metabolism, citrate cycle (TCA cycle), and flavonoid biosynthesis pathways were the smallest.

### 2.10. Transcription Factor Classification of Differentially Expressed Genes

**Figure 8:**
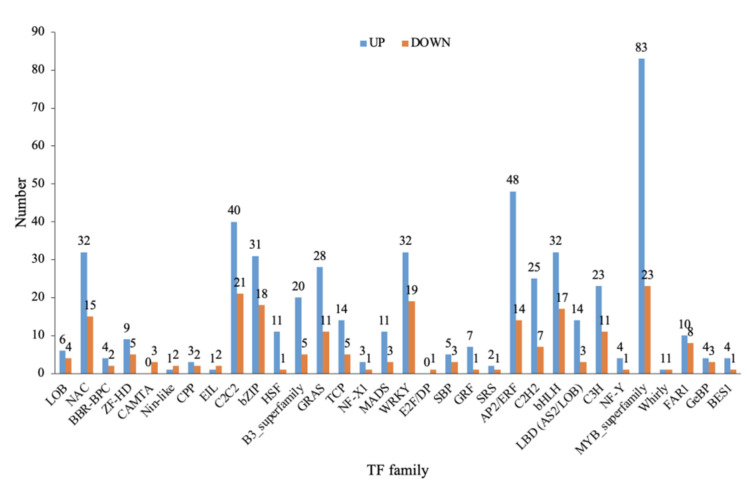
Differentially expressed transcription factors between T9 and CK.

The differentially expressed transcription factors were analyzed, as shown in Figure 8. Overall, there were 722 differentially expressed transcription genes in the treatment group, involving 32 types of transcription factor families: LOB (Up: 6, 60%; Down: 4, 40%), NAC (Up: 32, 68.0%; Down: 15, 32%), BBR-BPC (Up: 4, 66.7%; Down: 2, 33.3%), ZF-HD (UP: 9, 64.2%; Down: 5, 35.8%), CAMTA (Up: 0, 0%; Down: 3, 100%), Nin-like (Up: 1, 33.4%; Down: 2, 66.6%), CPP (Up: 3, 60%; Down: 2, 40%), EIL (Up: 1, 33.4%; Down: 2, 66.6%), C2C2 (Up: 40, 65.5%; Down: 21, 34.5%), bZIP (Up: 31, 63.2%; Down: 18, 36.7%), HSF (Up: 11, 91.6%; Down: 1, 8.3%), B3-superfamily (Up: 20, 80%; Down: 5, 20%), GEAS (Up: 28, 71.7%; Down: 11, 28.2%), TCP (Up: 14, 73.6%; Down: 5, 26.3%), NF-X1 (Up: 3, 75%; Down: 1, 25%), MADS (Up: 11, 78.5%; Down: 3, 21.5%), WRKY (Up: 32, 62.7%; Down: 19, 37.3%), E2F/DP (Up: 0, 0%; Down: 1, 100%), SBP (Up: 5, 62.5%; Down: 3, 37.5%), GRF (Up: 7, 87.5%; Down: 1, 12.5%), SRS (Up: 2, 66.7%; Down: 1, 33.3%), AP2/ERF (Up: 48, 77.4%; Down: 14, 22.6%), C2H2 (Up: 25, 78.2%; Down: 7, 21.8%), bHLH (Up: 32, 65.3%; Down: 17, 34.7%), LBD (AS2/LOB) (Up: 14, 82.3%; Down: 3, 17.7%), C3H (Up: 23, 67.7%; Down: 11, 32.3%), NF-Y (Up: 4, 80%; Down: 1, 20%), MYB-Superfamily (Up: 83, 78.3%; Down: 23, 21.7%), Whirly (Up: 1, 50%; Down: 1, 50%), FAR1 (Up: 10, 55.7%; Down: 8, 44.4%), GeBp (Up: 4, 57.2%; Down: 3, 42.8%), Bes1 (Up: 4, 80%; Down: 1, 20%). Among them, the MYB superfamily had the most transcription factors, up to 106, followed by AP2/ERF and C2C2, with 62 and 61 transcription factors, respectively. These were followed by WRKY, bZIP, bHLH, and NAC, among which the GEF, SBP, LOB, HSF, and MADS family transcription factors were the fewest.

### 2.11. Key Gene Analysis

**Figure 9:**
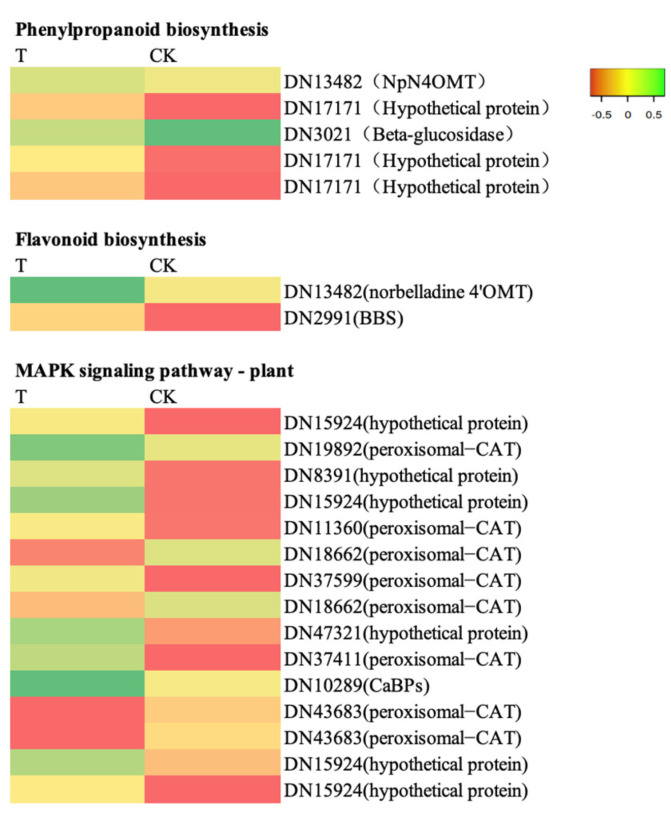
Heatmap of differential gene expression in the T9 treatment.

As shown in Figure 9, there were fifteen differentially expressed genes (eight *CAT*, one *CaBP,* and six hypothetical proteins) in the MAPK signaling pathway. Among them, four *CAT* genes were significantly downregulated, and the rest were significantly upregulated. These fifteen genes may be the key genes through which the T9 treatment affected *D. denneanum* growth. As shown in Figure 9, there were five differentially expressed genes (one *NpN4OMT*, one *β-glucosidase,* and three *hypothetical proteins*) in the phenylpropane biosynthesis pathway. β-glucosidase was downregulated and the hypothetical proteins and *NpN4OMT* were upregulated. These genes may be key genes through which the T9 treatment affected the accumulation of polysaccharides and flavonoids. Two genes (one *NpN4OMT* and one *BBS*) in the flavonoid biosynthesis pathway were significantly upregulated, indicating that they were involved in the biosynthesis of flavonoids in *D. denneanum*.

### 2.12. RT-qPCR Analysis

The expression levels of DN13482 (norbelladine 4′OMT), DN2991 (BBS), DN17171 (hypothetical protein), DN3021 (beta-glucosidase), DN15924 (hypothetical protein), DN19892 (peroxisomal−CAT), DN8391 (hypothetical protein), DN11360 (peroxisomal−CAT), and DN37599 (peroxisomal−CAT) were verified by RT-qPCR. As shown in Figure S2, the expression levels of the nine differential genes were basically consistent with the changes in transcriptome sequencing.

## 3. Discussion

### 3.1. Effects of N, P, and K on the Growth and Main Medicinal Compounds of D. denneanum

Antioxidant enzyme activity is closely related to the age of plant leaves [21]. In this experiment, the SOD and POD activities were the highest in the medium (N2) and high (N3) nitrogen concentrations at 60 d, which indicated that the concentration of nitrogen in the fertilizer had a strong effect on promoting antioxidant enzyme activity. The difference in CAT activity between the T9 treatment and the control was the most significant, indicating that high nitrogen and phosphorus promoted CAT activity in *D. denneanum*. These results indicate that higher N concentrations may be conducive to improving the activity of protective enzymes, thus making *D. denneanum* more resistant to aging, which is conducive to the growth (Figure S3).

Flavonoids and polysaccharides are the main medicinal compounds in *D. denneanum* [22] and their content is an important index to measure the quality of this species. In this experiment, the polysaccharide contents of plants treated with T2, T3, T4, T6, T7, T8, and T9 were significantly higher than the control, which showed that spraying appropriate amounts of nitrogen, phosphorus, and potassium fertilizer promoted the polysaccharide content of *D. denneanum*. The increase in polysaccharides was the highest in the T9 group; therefore, 1500 mg·L^−1^ nitrogen and 3000 mg·L^−1^ phosphorous had the strongest promoting effect on the polysaccharide content. All treatment concentrations significantly increased the flavonoid content in plants, indicating that fertilizing with nitrogen, phosphorus, and potassium increased the flavonoid content of *D. denneanum*.

### 3.2. Effects of T9 Treatment on Gene Expression and Biosynthesis Pathways

Transcriptome sequencing can be used to identify functional genes and metabolic pathways in medicinal plants to determine key enzyme genes in the secondary metabolism biosynthesis pathways. Moreover, transcriptome sequencing provides a molecular basis for the collection and evaluation of medicinal plant germplasm resources, the identification, preservation, and expansion of germplasm resources, and the breeding of fine germplasm [23]. For example, Li et al. [24] used strain *MF23* to impregnate tissue culture seedlings of *D. nobile* and screened 30 genes related to dendrobine synthesis through transcriptome sequencing. Wen et al. [25] used transcriptome sequencing to screen genes related to flower bud differentiation in *D. nobile*. The results of high-throughput transcriptome sequencing can reveal the overall expression characteristics of the transcriptome during plant growth and development. The results can be used to rapidly interpret the genomic information of a species, and thus mine the functional genes [26]. Using the GO and KEGG databases, 2277 differentially expressed genes (1230 upregulated and 1047 downregulated) were found to be related to signal transduction mechanisms in the transcriptome of *D. denneanum* leaves. Fifteen genes were in the MAPK signaling pathway, two were related to flavonoid biosynthesis, and five were related to phenylpropanoid biosynthesis. The MAPK signaling system plays an important role in plant growth, development, disease resistance, and other physiological processes, such as the stress response [27]. In the T9 treatment, eleven genes in the MAPK signaling pathway were upregulated, and four were downregulated. The POD, CAT, soluble sugar, and chlorophyll levels were significantly higher in the T9 group than in the other treatment groups; therefore, *D. denneanum* growth might be regulated by the MAPK signaling pathway. Additionally, five genes related to phenoloid biosynthesis and two genes related to flavonoid biosynthesis were significantly upregulated in the T9 group. Phenoloid biosynthesis is involved in polysaccharide biosynthesis, and two genes related to flavonoid biosynthesis were identified in *D. denneanum*. In the T9 treatment, the polysaccharide content was significantly higher than the other groups, and the flavonoid content was significantly higher than the control group. Therefore, it can be inferred that the biosynthesis of polysaccharides and flavonoids in *D. denneanum* is regulated by the five upregulated phenylpropanoid biosynthesis genes and the two flavonoid biosynthesis genes.

### 3.3. Effect of T9 Treatment on Transcription Factors

Transcription factors play an important role in regulating the accumulation of secondary metabolites in plants. MYB and NAC families activate the expression of genes related to the synthesis of cell wall components and the regulation of cell wall synthesis. MYBs play an important role in regulating the expression of structural genes of multiple secondary metabolite pathways. For example, *AtMYB12* is a key gene that affects the flavonoid biosynthesis pathway and caffeoylquinic acid biosynthesis pathway, leading to the massive accumulation of these two polyphenols in tomatoes [28]. In *Lilies*, the *LrMYB15* gene was found to stop transcription under shade conditions, and the color of flower buds became pale, indicating that the transcription of this gene was controlled by light [29]. Ma et al. [30] found that *li049* gene knockout in AP2/ERF members decreased the gene transcription level in the lignin biosynthesis pathway, proving that the *li049* gene plays an important role in lignin biosynthesis. In the present study, we found that most of the MYB and AP2/ERF transcription factors were significantly upregulated, which may be the reason for the significant upregulation of gene expression levels, such as for hypothetical protein and *NpN4OMT* in the phenylpropanoid biosynthesis pathway. Under the T9 treatment, plant growth, the polysaccharide content, and the flavonoid content of *D. denneanum* were significantly higher than the control group, indicating that these transcription factors may regulate *D. denneanum* growth and the synthesis of its main medicinal components.

## 4. Materials and Methods

### 4.1. Plant Material and Treatments

Annual *D. denneanum* seedlings with a plant height of 10.0 ± 2.0 cm were planted in pots with an inner diameter of 30 cm and a height of 25 cm. There were 20 plants in each pot and 6 pots per group. The matrix ratio of planting was pine bark: ceramsite: stone = 2:1:1, with 75% shading (75% shade net).

According to the L_9_ (3^4^) orthogonal experimental design (Table 4), the plants were sprayed with different concentrations of nitrogen (CH_4_N_2_O), phosphorus (Ca (H_2_PO_4_)_2_ H_2_O), and potassium (K_2_SO_4_) and the same amount of distilled water was sprayed as the control. The first spraying was started 1 week after transplanting and colonization and was conducted once every 7 days. Spraying was applied from 7:00 to 10:00 in the morning. The sprayed amount was carried out in order that both sides of each leaf of each plant were wet, but the liquid on the leaf surface was not dripping. The sprayed liquid contained N, P, and K and trace elements were supplemented at the same time. During the experiment, the plants were routinely maintained by watering every 3 days, and plant growth was recorded. After that treatment, samples were taken every 30 days. For each sampling, 12 plants were randomly selected from each treatment to analyze the relevant indices.

### 4.2. Determination of Morphological Indices

The growth indices of *D. denneanum* included leaf width, the number of leaves, plant height, leaf length, and stem diameter. The plant height and stem diameter of each plant were measured by a five-point sampling method. The fourth leaf from the bottom was selected; the leaf length and width were measured, and the maximum leaf area was calculated (maximum leaf area = leaf length × leaf width × 0.78).

### 4.3. Determination of Physiological Indices and the Contents of the Main Medicinal Compounds

The soluble protein content was determined by the method of Li et al. [31], the soluble sugar content was determined by the phenol concentrated sulfuric acid method [32], and the chlorophyll content was determined by the acetone method [33]. SOD activity was determined by the method of Milosevic et al. [34], POD activity was determined by the guaiacol method [35], and CAT activity was determined by UV spectrophotometry [36]. The flavonoid content was determined by the colorimetric method of “sodium nitrite-aluminum nitrate-sodium hydroxide”, with rutin as the standard [37], and the polysaccharide content was determined by the phenol concentrated-sulfuric acid method, with anhydrous glucose as the standard [38].

### 4.4. Illumina Sequencing

Total RNA was extracted from *D. denneanum* according to the instructions of the RNA extraction kit. Using 5.0 μL of total RNA, the purity of RNA was detected by a Nanodrop 2000, and the integrity of RNA was detected by agarose gel electrophoresis. An Agilent 2100 was used to measure the RIN value. The RNA parameters required to establish a single library were ≥1.0 μg, a concentration ≥ 35.0 ng/μL, an OD260/280 ≥ 1.8, and an OD260/230 ≥ 1.0. RNA that met these requirements was used for cDNA library construction. Transcriptome sequencing was performed using Illumina HiSeq 2100 instrument (Shanghai Meiji Biological Company, Shanghai, China).

### 4.5. Transcriptome Data Processing and Analysis

The cDNA library was constructed according to the process of transcriptome library construction (no reference genome). After sequencing, de novo transcripts were assembled. The connector was removed from the raw data, and low-quality data were removed to obtain clean data. The sequencing quality of the samples was evaluated, and subsequent analyses were carried out only after the sequencing quality met the requirements. The transcripts were assembled with Trinity software (V2.4.0), and the CD-Hit program filtered out the low-quality transcripts. After removing redundancies, transcripts with FPKM >2 in at least one sample were retained as reliable transcripts.

The assembled unigene was annotated using seven functional databases (GO, KOG, KEGG, NR, NT, SwissProt, and InterPro) [39,40]. Blastn was used to annotate the unigene with NT, while Blastx and Diamond were used to annotate the unigene with KOG, KEGG NR, and SwissProt. The Blast2GO and NR annotation results were used to annotate GO, and InterProScan5 was used to annotate InterPro [41,42]. Bowtie2 [43] was used to compare clean reads to the unigene according to the assembly results, and RSEM [44] was then used to calculate the gene expression levels in each sample. The expression amount in each sample was standardized, and the differences were compared and analyzed using two groups of standardized data. The differentially expressed genes were assessed according to the fold change in gene expression and the *q*-value. Genes with *q* < 0.05 and |log_2_FC| > l (FC, fold change) were defined as differentially expressed genes.

### 4.6. qRT-PCR

DN15924 (hypothetical protein), DN19892 (peroxisomal-CAT), DN8391 (hypothetical protein), DN11360 (peroxisomal-CAT), and DN37599 (peroxisomal-CAT) from MAPK signaling pathway; DN13482 (norbelladine 4′OMT) and DN2991 (BBS) from phenylpropane biosynthesis pathway; DN17171 (hypothetical protein) and DN3021 (beta-glucosidase) from flavonoid biosynthesis pathway were randomly selected to verify the accuracy of transcriptome analysis. The DN18662 gene was set as an internal standard to calculate relative fold differences based on the comparative cycle threshold ($2^{-\Delta \Delta \mathrm{Ct}}$) method. The primer sequences were designed by Primer premier 6.0 and listed in Table S3. Total RNA of *D. denneanum* was extracted and reverse transcribed to synthesize cDNA samples for 3 biological repetitions.

### 4.7. Data Analysis

SPSS 27 was used to analyze significant differences. Duncan’s method was used to assess the significance level of the data at *p* = 0.05, and one-way ANOVA, Pearson correlation analysis, and principal component analysis (KMO and Bartlett’s sphericity test) were used to test the applicability.

## 5. Conclusions

In this study, nine different levels of nitrogen, phosphorus, and potassium foliar fertilizers were applied to *D. denneanum* using an L_9_ (3^4^) orthogonal experimental design. The morphological indices and chlorophyll content of the plant were positively correlated with the nitrogen concentration. The application of 1500 mg·L^−1^ nitrogen and 3000 mg·L^−1^ phosphorus had the strongest effect on promoting the polysaccharide content. Under the same nitrogen concentration, the flavonoid content was the highest at 500 mg·L^−1^ potassium. The principal component score analysis showed that nitrogen had the most significant impact on the various indicators of *D. denneanum*, followed by phosphorus and potassium. The comprehensive score showed that the T9 treatment (1500 mg·L^−1^ nitrogen, 3000 mg·L^−1^ phosphorus, and 500 mg·L^−1^ potassium) had the strongest effect. 

Transcriptome analysis showed that in the T9 treatment group, the differentially expressed genes were mainly enriched in the ribosome, tryptophan metabolism, glyceroid metabolism, oxidative photosynthesis, biosynthesis of factors, glycolysis/gluconeogenesis, and other pathways. Among them, the MAPK signaling pathway, phenylpropanoid biosynthesis, and flavonoid biosynthesis might regulate the growth and medicinal components of *D. denneanum*. Additionally, most MYB and AP2/ERF transcription factors in the T9 treatment were significantly upregulated, which may be the reason for the significantly upregulated expression of genes, such as hypothetical proteins and *NpN4OMT* in the phenylpropanoid biosynthesis pathway.

## Figures and Tables

**Table 1:** Effects of different levels of nitrogen, phosphorus, and potassium on chlorophyll A and chlorophyll B contents.

Chlorophyll	Treatment	30 d	60 d	90 d
		(mg·g^−1^ FW)	(mg·g^−1^ FW)	(mg·g^−1^ FW)
Chl. A	T1	0.46 ± 0.01 de	0.75 ± 0.01 d	0.82 ± 0.03 c
	T2	0.51 ± 0.02 c	0.83 ± 0.02 b	0.89 ± 0.01 a
	T3	0.43 ± 0.02 f	0.75 ± 0.01 d	0.78 ± 0.03 d
	T4	0.49 ± 0.02 cd	0.77 ± 0.01 c	0.84 ± 0.01 c
	T5	0.52 ± 0.01 bc	0.81 ± 0.02 b	0.86 ± 0.02 b
	T6	0.49 ± 0.04 bc	0.75 ± 0.03 d	0.79 ± 0.02 d
	T7	0.57 ± 0.02 a	0.86 ± 0.01 a	0.90 ± 0.03 a
	T8	0.55 ± 0.02 ab	0.85 ± 0.02 a	0.91 ± 0.02 a
	T9	0.56 ± 0.03 a	0.87 ± 0.02 a	0.90 ± 0.04 a
	CK	0.45 ± 0.02 ef	0.72 ± 0.03 e	0.74 ± 0.05 e
Chl. B	T1	0.24 ± 0.02 de	0.38 ± 0.01 f	0.40 ± 0.02 e
	T2	0.26 ± 0.03 b	0.45 ± 0.02 ab	0.46 ± 0.01 ab
	T3	0.22 ± 0.02 f	0.39 ± 0.01 f	0.42 ± 0.04 cde
	T4	0.25 ± 0.01 bc	0.43 ± 0.02 ab	0.44 ± 0.02 bc
	T5	0.30 ± 0.02 a	0.41 ± 0.01 cd	0.43 ± 0.03 cd
	T6	0.25 ± 0.01 cd	0.41 ± 0.02 de	0.42 ± 0.01 cde
	T7	0.29 ± 0.03 a	0.45 ± 0.01 a	0.44 ± 0.05 bc
	T8	0.29 ± 0.02 a	0.43 ± 0.01 bc	0.47 ± 0.03 a
	T9	0.30 ± 0.02 a	0.44 ± 0.02 ab	0.46 ± 0.02 ab
	CK	0.23 ± 0.02 ef	0.39 ± 0.02 ef	0.40 ± 0.02 e

Note: Lowercase letters indicate inter group significance (*p* < 0.05).

**Table 2:** Correlation analysis of the morphology, physiology, and main medicinal compounds of *D. denneanum* at 90 d (* *p* < 0.05, ** *p* < 0.01).

	Number of Blades	Maximum Leaf Area	Plant Height	Stem Diameter	Chl. A	Chl. B	Soluble Protein	Soluble Sugar	SOD	POD	CAT	Polysaccharides	Flavonoids
Number of blades	1												
Maximum leaf area	0.320	1											
Plant height	0.720 *	0.847 **	1										
Stem diameter	0.472	0.67 1*	0.772 **	1									
Chl. A	0.398	0.898 **	0.878 **	0.805**	1								
Chl. B	0.331	0.894 **	0.776 **	0.578	0.865 **	1							
Soluble protein	0.290	0.708 *	0.656 *	0.537	0.737 *	0.734 *	1						
Soluble sugar	0.046	0.832 **	0.569	0.440	0.755 *	0.646 *	0.676 *	1					
SOD	0.179	0.504	0.360	0.458	0.640 *	0.531	0.505	0.670 *	1				
POD	0.331	0.638*	0.599	0.745 *	0.722 *	0.724 *	0.749 *	0.468	0.624	1			
CAT	−0.066	0.556	0.266	0.179	0.430	0.473	0.353	0.719 *	0.537	0.471	1		
Polysaccharides	0.168	0.511	0.342	0.476	0.447	0.582	0.693*	0.476	0.559	0.835 **	0.577	1	
Flavonoids	0.776 **	0.579	0.748 *	0.692 *	0.594	0.472	0.403	0.390	0.463	0.406	0.021	0.335	1

**Table 3:** Principal component scores, comprehensive scores, and rankings of different treatments.

Treatments	Components		Comprehensive Score	Ranking
	1	2		
T1	−1.004	−1.736	−0.834	9
T2	0.527	−1.367	0.151	4
T3	−0.634	0.460	−0.331	7
T4	0.233	−0.281	0.108	5
T5	−0.485	−0.320	−0.338	8
T6	−0.073	0.688	0.0421	6
T7	1.070	0.254	0.688	2
T8	0.899	−0.211	0.524	3
T9	1.301	1.197	0.949	1
CK	−1.834	1.312	−0.959	10

**Table 4:** Concentrations of nitrogen, phosphorus, and potassium in each treatment.

Treatment	N(CH_4_N_2_O) mg·L^−1^	P(Ca(H_2_PO_4_)_2_·H_2_O) mg·L^−1^	K(K_2_SO_4_) mg·L^−1^
T1 (N1P1K1)	500	1000	300
T2 (N1P2K2)	500	2000	500
T3 (N1P3K3)	500	3000	700
T4 (N2P1K2)	1000	1000	500
T5 (N2P2K3)	1000	2000	700
T6 (N2P3K1)	1000	3000	300
T7 (N3P1K3)	1500	1000	700
T8 (N3P2K1)	1500	2000	300
T9 (N3P3K2)	1500	3000	500
CK	0	0	0

## Data Availability

Not applicable.

## Supplementary Materials

The following supporting information can be downloaded at: https://www.mdpi.com/article/10.3390/ijms24021522/s1.
