# Supplementary material for: Transcriptional and Physiological Analysis Reveal New Insights into the Regulation of Fertilization (N, P, K) on the Growth and Synthesis of Medicinal Components of Dendrobium denneanum
Source: Int J Mol Sci. 2023 Jan 12;24(2):1522. doi: 10.3390/ijms24021522 (PMC9866100; doi:10.3390/ijms24021522)
Supplement: Supplementary file 1 [file ijms-24-01522-s001.zip › ijms-2134480-supplementary.pdf]

Supplementary Data

Table S1. Component score coefficient matrix.

Index	Components	
	1	2
Chlorophyll A	0.159	-0.267
Chlorophyll B	0.161	-0.050
Soluble protein	0.154	-0.142
Soluble sugar	0.151	0.150
SOD	0.141	0.025
POD	0.153	0.011
CAT	0.116	0.605
Polysaccharide	0.128	0.259
Flavonoid	0.102	-0.575

Table S2. Total RNA sequencing yield statistics of each sample

Sample ID	Raw reads	Raw bases	Clean reads	Clean bases	Error rate/%	Q20/%	Q30/%	GC content/%
T3	62833360	9487837360	59795974	8768816680	0.0254	97.8	93.78	45.14
T2	59992278	9058833978	57624978	8506432931	0.0257	97.71	93.48	44.75
T1	64862694	9794266794	62094754	9082763361	0.0253	97.84	93.91	45.29
CK3	77801192	11747979992	76071324	11069845003	0.0249	98.01	94.24	45.76
CK2	61891962	9345686262	59195792	8635910319	0.0259	97.65	93.35	45.09
CK1	78606696	11869611096	75311874	10894259484	0.0251	97.95	94.14	45.21
Total	405988182	61304215482	390094696	56958027778				

Table S3. Primers used in this study for qRT-PCR assay

Genes_id	Primer	Sequence
DN18662	Forward primer	CTCGACCATGTTTTGGGACTA
	Reverse primer	TCTGAACGTTCTCGTTGAGGT
DN37599	Forward primer	GCGTACAAATTCGGCAACT
	Reverse primer	GAACTGCATATCCTTGATGCC
DN11360	Forward primer	ACAGCAAGGGGTAAAGTGGAT
	Reverse primer	GCAGTTGCTGGTAATTGGTTC
DN8391	Forward primer	GCTGGTGAATAAGGATGGTGA
	Reverse primer	AACAGTCGAGACTGGAGGACA
DN19892	Forward primer	ACATGAAGAGCAAGCAGGGTA
	Reverse primer	CCCTGGTTGTAGAATGCCATA
DN15924	Forward primer	CAACGTCTTCGACTTGACACA
	Reverse primer	GCATTGAAGTCCTCTGGTCTG
DN3021	Forward primer	CCGCGTATAGGAAGAGGAACT
	Reverse primer	CAATACTTGACCCTATCGCCA
DN17171	Forward primer	CAAGACATCTCTGCCCTCAAG
	Reverse primer	TCTTGAGTTGGCTCCAAGTGT
DN2991	Forward primer	TGCTCTATCAGCAGGGTTGTT
	Reverse primer	CACCTGATCTAGAATGGCTCG
DN13482	Forward primer	GTACATTCAGAAAGCTGGCGT
	Reverse primer	TCCTATGATCCGAGGCTAATTC

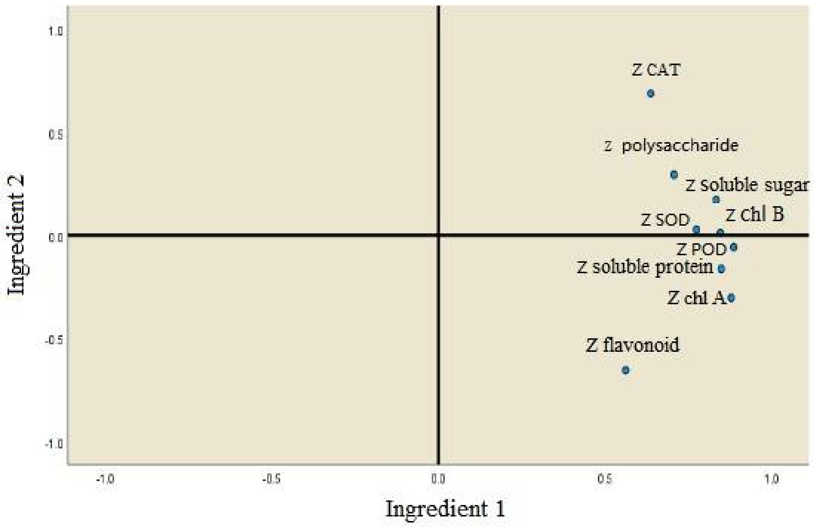

Figure S1. Factor of loading matrix

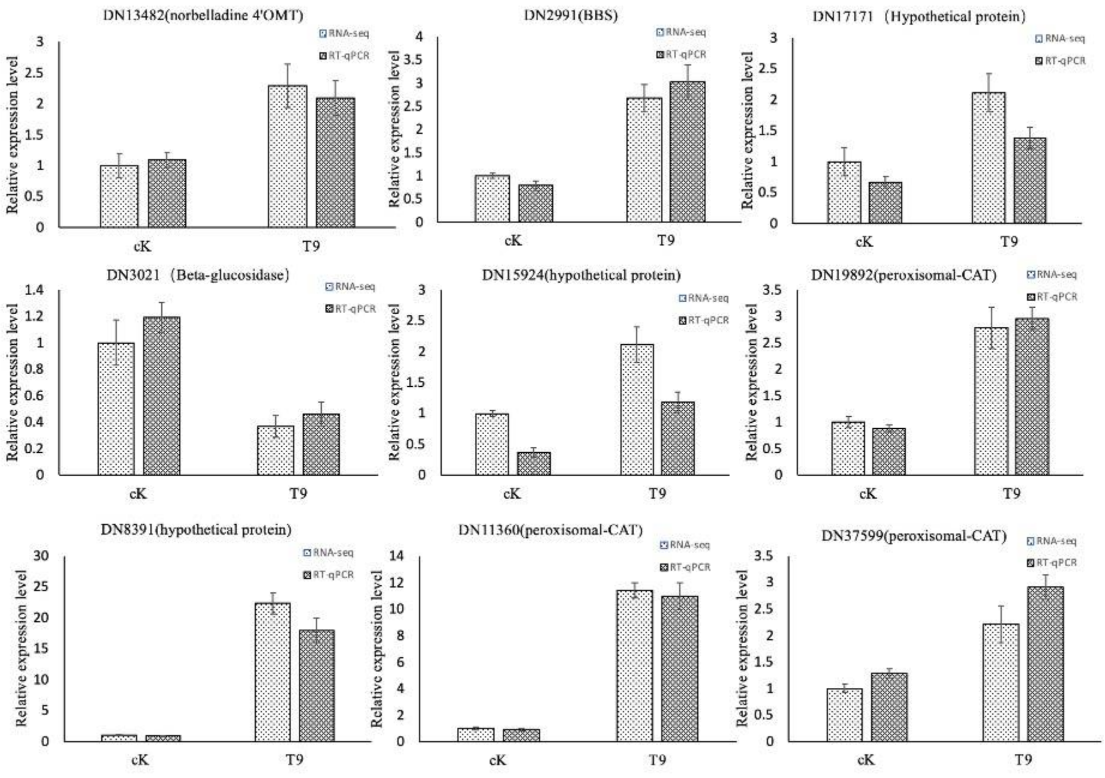

Figure. S2. qRT-PCR validation of candidate genes related to T9 treatment regulating D. denneanum.

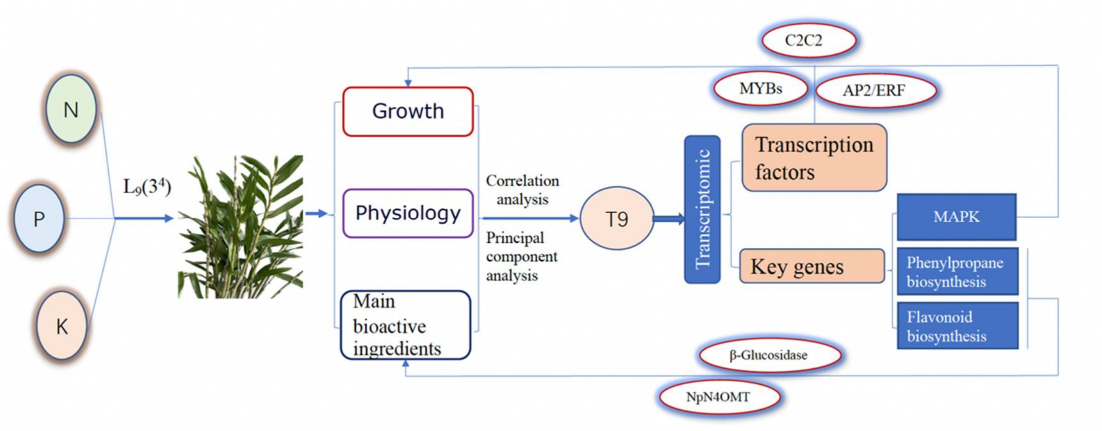

Figure S3. Physiological and transcriptomic analyses of the response of *D. denneanum* to different levels of nitrogen, phosphorus and potassium
